# A rare pediatric tumor: thymic carcinoma mimicking acute rheumatoid fever

**DOI:** 10.1186/1546-0096-12-S1-P145

**Published:** 2014-09-17

**Authors:** Muferet Erguven, Asim Yoruk, Olcay Yasa, Mehpare Yanartas, Sebnem Batur, Buge Oz

**Affiliations:** 1 Department of Pediatrics, Ministry of Health, Istanbul Medeniyet University Goztepe Research, Istanbul, Turkey; 2 Department of Pediatric Hematology and Oncology, Ministry of Health, Istanbul Medeniyet University Goztepe Research and Training Hospital, Istanbul, Turkey; 3 Department of Pathology, Istanbul University, Cerrahpasa Medical Faculty, İstanbul, Turkey

## Introduction

Malign diseases may mimic rheumatoid diseases. Joint involvement and leg pain are among frequently encountered symptoms particularly in the patients with leukemia. Sometimes, however, primary tumor may be asymptomatic and may mimic rheumatoid diseases because of metastasis. The present case, which admitted to our clinic with bone and joint pain continuing for 10 days and mimicking acute rheumatoid fever (ARF) and in which bone metastasis due to thymic carcinoma was detected when investigated, was presented for it is a rare condition.

## Objectives

The present case, which admitted to our clinic with bone and joint pain continuing for 10 days and mimicking acute rheumatoid fever (ARF) and in which bone metastasis due to thymic carcinoma was detected when investigated, was presented for it is a rare condition.

## Methods

A 13-year-old male patient presented to the pediatric policlinic with joint swelling and leg pain that appeared 10 days ago. His history revealed that pain wakes him up at night and is remittent, spreads over his thigh and accompanied by knee pain, and that he has no history of recent infection or trauma.

The results of the laboratory analyses were as follows: erythrocyte sedimentation rate 63 mm/hour, C-reactive protein 3.57 mg/dl and ASO 358 IU, and the patient was considered as ARF and admitted to the clinic for further analysis. Joint examination revealed swelling, but had no warmness or redness. Results of the analyses performed for joint pain including rheumatoid factor, anti-nuclear antibody, anti-dsDNA and other markers of collagen vascular diseases, as well as brucella and salmonella agglutination tests, were all negative. Bone marrow aspiration was normal.

MRI of hip, knee and sacroiliac joint were performed. Metastases were detected in all pelvic bones being more prominent in the left side including femur neck and proximal diaphysis, in bilateral sacrum and iliac bone with the largest was 28mm in the left iliac bone; multiple round-shaped metastases located in the femur, distal tibia and proximal diaphysis with the largest was 1 cm.

Contrast enhanced thoracic CT demonstrated an approximately 10 x 7.5 x 5 cm heterogeneous hypodense solid-like mass lesion in the anterior mediastinum.

On PET/CT, increased FDG uptake was observed at malignancy level with a SUVmax measured to be 7 in a 76 x 46 x 49 mm mass, which completely fills the anterior mediastinum, suppresses the left lung, pushes the mediastinal structures towards to the right and posterior, and invades the sternum. And there were increased FDG uptake in various areas of the lungs.

Increased FDG uptake at malignancy level with SUVmax measured to be 8.3 in bilateral humerus, bilateral scapula, sternum, extensively in C3, C7, thoracic and lumbar vertebras, in the right 8^th^ and left 5^th^ and 6^th^ costae, sacrum, bilateral iliac, acetabulum, head and neck of femur, pubis, and ischium.

## Results

The result of the biopsy taken from the mass with the assistance of interventional radiology came up as thymic carcinoma.

## Conclusion

The present case, which presented with the prediagnosis of ARF, is interesting because of having thymic carcinoma, is a rare condition in childhood, and no complaint other than leg pain for 10 days was present despite extensive metastasis at the time of presentation.

## Disclosure of interest

None declared.

